# ORegAnno 3.0: a community-driven resource for curated regulatory annotation

**DOI:** 10.1093/nar/gkv1203

**Published:** 2015-11-17

**Authors:** Robert Lesurf, Kelsy C. Cotto, Grace Wang, Malachi Griffith, Katayoon Kasaian, Steven J. M. Jones, Stephen B. Montgomery, Obi L. Griffith, The Open Regulatory Annotation Consortium

**Affiliations:** 1 McDonnell Genome Institute, Washington University School of Medicine, St. Louis, MO 63108, USA; 2 Department of Genetics, Washington University School of Medicine, St. Louis, MO 63110, USA; 3 Siteman Cancer Center, Washington University School of Medicine, St. Louis, MO 63110, USA; 4 Canada's Michael Smith Genome Sciences Centre, BC Cancer Agency, Vancouver, BC V5Z 4S6, Canada; 5 Department of Molecular Biology & Biochemistry, Simon Fraser University, Burnaby, BC V5A 1S6, Canada; 6 Department of Medical Genetics, University of British Columbia, Vancouver, BC V6T 1Z3, Canada; 7 Department of Pathology, Stanford University School of Medicine, Stanford, CA 94305, USA; 8 Department of Genetics, Stanford University School of Medicine, Stanford, CA 94305, USA; 9 Department of Medicine, Division of Oncology, Washington University School of Medicine, St. Louis, MO 63110, USA

## Abstract

The Open Regulatory Annotation database (ORegAnno) is a resource for curated regulatory annotation. It contains information about regulatory regions, transcription factor binding sites, RNA binding sites, regulatory variants, haplotypes, and other regulatory elements. ORegAnno differentiates itself from other regulatory resources by facilitating crowd-sourced interpretation and annotation of regulatory observations from the literature and highly curated resources. It contains a comprehensive annotation scheme that aims to describe both the elements and outcomes of regulatory events. Moreover, ORegAnno assembles these disparate data sources and annotations into a single, high quality catalogue of curated regulatory information. The current release is an update of the database previously featured in the NAR Database Issue, and now contains 1 948 307 records, across 18 species, with a combined coverage of 334 215 080 bp. Complete records, annotation, and other associated data are available for browsing and download at http://www.oreganno.org/.

## INTRODUCTION

The Open Regulatory Annotation database (ORegAnno) was first released about a decade ago (1), with the intention to collect and synthesize a catalogue of regulatory elements. It remains unique in the field because of its focus on collecting high quality, curated regulatory records from the literature. Moreover, ORegAnno relies on a thriving community of scientists who are interested in contributing to the resource, as well as utilizing its data. Since the last release of ORegAnno in early 2008 (2), the amount and types of published regulatory data have grown exponentially. This relates in part to high-throughput studies from the ENCODE consortium and others, who have performed an enormous number of ChIP-seq, DNase-seq, FAIRE-seq and other experiments aiming to identify biochemically available and transcriptionally active regions of genomes (3). While these efforts are excellent resources for identifying candidate regulatory regions, ENCODE efforts have suggested that as much as 80% of the genome could be functional (3). This controversial finding has been the focus of much attention in the community, with several commentaries pointing out that these types of high-throughput data are prone to overestimates due to experimental and statistical methods that result in a high number of false positive calls (4–6). Moreover, they do not necessarily provide a comprehensive understanding of all of the elements involved in gene regulation. For example, knowing the region of DNA that is bound by a transcription factor does not directly indicate whether the expression of any genes are altered, nor whether an alteration results in up- versus down-regulation. Validation of the genomic regions identified by ENCODE and others requires a large number of low-throughput experimental data paired with careful manual curation. Additionally, much of the available evidence supporting gene regulation is dispersed across various experiments, specialized datasets, and individual publications, making it cumbersome to obtain regulatory information that has been released by the community across this broad set of sources. The current version of ORegAnno seeks to address these issues by cataloging a large number of new, curated, high quality regulatory records that are derived from published literature and other data resources.

## RESULTS

### Overview

**Figure 1.**
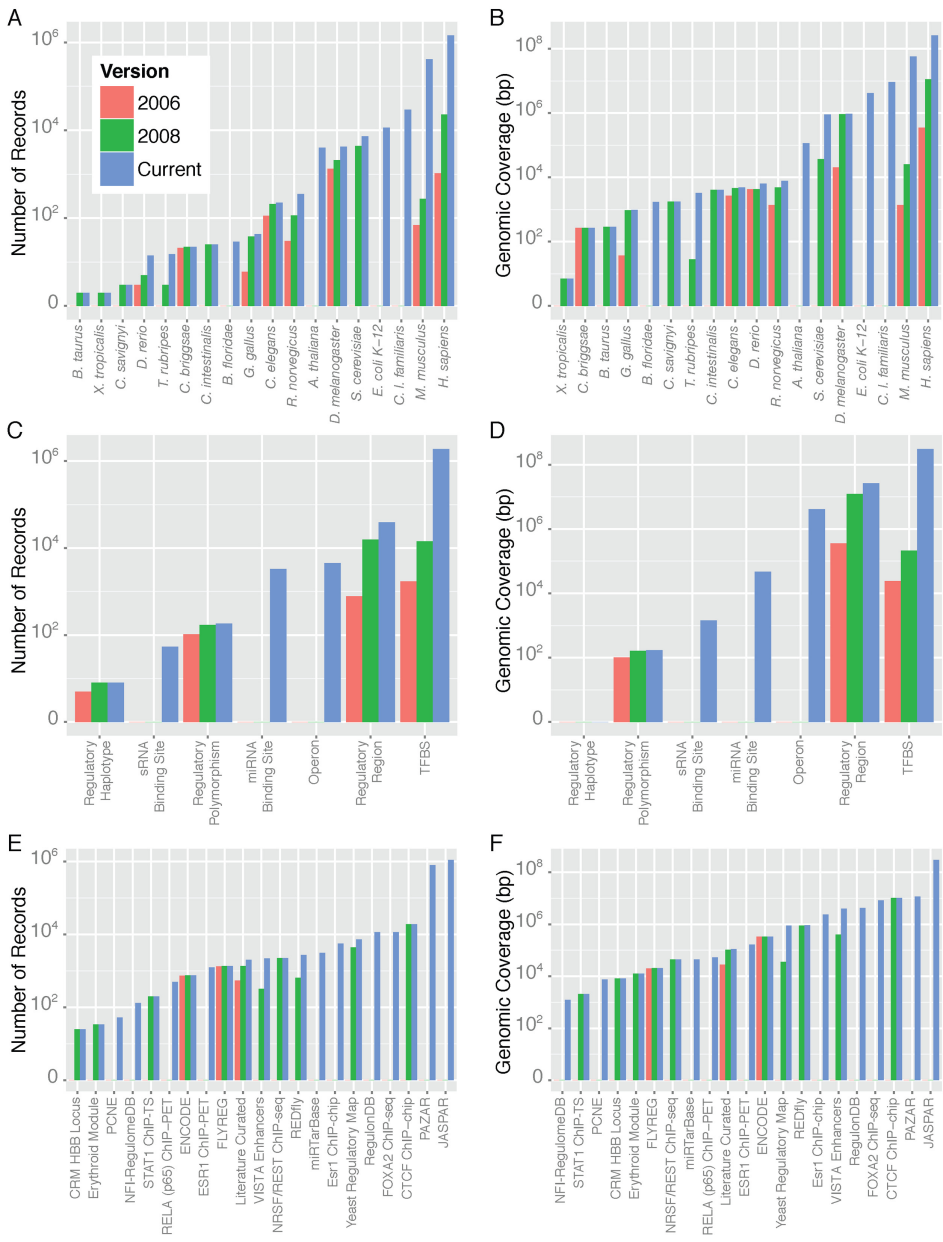
Current content of the ORegAnno database. Content statistics are divided by species (**A** and **B**), regulatory type (**C** and **D**), and data source (**E** and **F**).

The current version of ORegAnno now has a total of 1 948 307 unique records. These records cover a combined 334 215 080 bp across 18 species (Figure 1A and B). The vast majority of these records are mapped to human and mouse genomes, with 1 452 466 records in human (261 660 516 bp in the GRCh38/hg38 genome assembly version) and 415 808 records in mouse (57 253 973 bp in the GRCm38/mm10 genome assembly version).

As a measure of the success of our community-based participation, ORegAnno currently has 1044 registered users. Aside from the principal authors of this paper, 13 301 records have been contributed by members of the broader community (The Open Regulatory Annotation Consortium). ORegAnno continues to have a robust verification system to ensure that contributed records are accurate and appropriately annotated. A set of trusted consortium members have been granted a ‘validator’ status, allowing them to review and up- or down-vote records. This results in individual record scores that are visible to all users. Moreover, when a record is negatively scored, it will typically be assigned a deprecated status. ORegAnno additionally includes an ontology for summarizing the experimental evidence that supports the regulatory elements and outcome in each record. Together, these features allow users to filter records according to various quality criteria.

The ORegAnno database has served as a repository for publishing regulatory sites derived from experimental data (7), and it has been incorporated into other resources including the Babelomics (8), cisRED (9), ConTra (10), GRASP (11), i-cisTarget (12), LASAGNA-Search 2.0 (13), the UCSC Genome Browser (14) and more. Similarly, the annotated information included in ORegAnno has been used to construct gene regulation networks for the development of other tools and the analysis of gene expression data (15–19). ORegAnno records were used in the REC-set design for a capture sequence reagent (20), and as part of the definition for regulatory sites of the human genome (tier 2) in the Genome Modeling System (21), an analysis information management system at the McDonnell Genome Institute of Washington University that has been used to process over 4800 human whole genome samples, over 40 000 exomes, and over 1400 transcriptomes. Similarly, ORegAnno has been adapted into the information systems of other research centers including the Broad Institute and Cancer Research UK, where it has been used in the analysis of several high impact studies (22–25).

**Figure 2.**
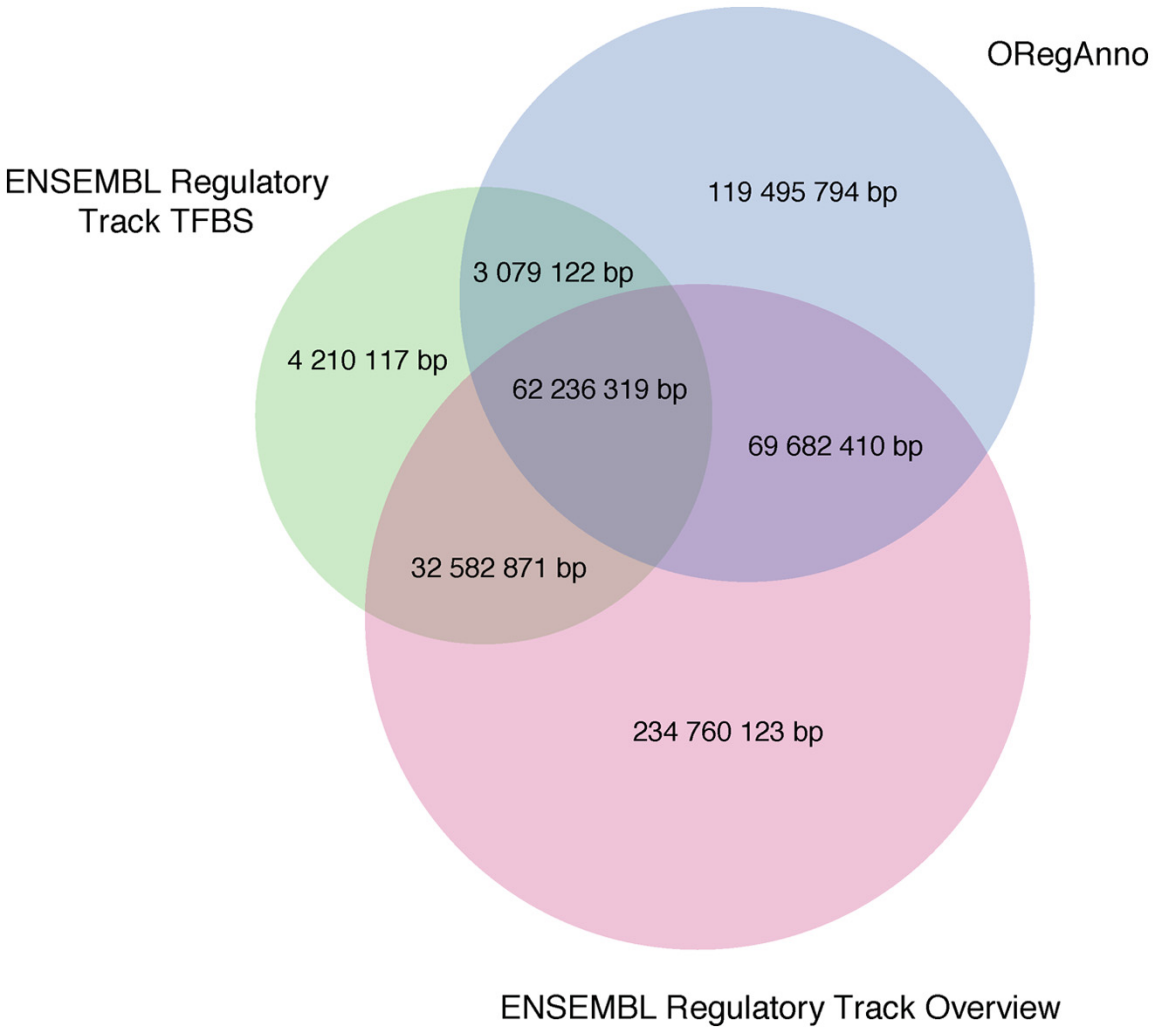
Comparison of the genomic coverage captured by ORegAnno and the ENSEMBL Regulatory Track. A Venn diagram demonstrates coverage overlaps for human genome assembly version GRCh38/hg38, with sets sized to scale. The ENSEMBL Regulatory Track is divided into two main sets, a track overview set and the transcription factor binding site (TFBS) set.

Because ORegAnno focuses on curated regulatory information, the total genomic coverage found in ORegAnno is smaller than that identified by resources such as ENCODE or the ENSEMBL regulatory tracks (26), which are largely a summary of ENCODE data (Figure 2). This trade off is part of an effort to ensure that ORegAnno represents a high-quality curated set of regulatory elements, with the aim of maintaining a low number of false positive records.

### Updates

Older records, including those that were added through crowd-sourcing efforts via the web, have been updated to ensure that only accurate and up-to-date gene symbols are being used. This was accomplished through a combination of automatically updating symbols using NCBI Gene or ENSEMBL identifiers, as well as by manually checking incorrect and missing data. In addition, previously missing identifiers from NCBI Gene or ENSEMBL have been added where possible, allowing for future automated updates to ensure the accuracy of these gene lists. These updates have resulted in 423 automated changes and 13 174 manually curated changes (13 597 total) affecting 10 386 records.

For all ORegAnno records (existing and new), genomic coordinates have been updated and expanded using liftOver (27). This involved converting older genomic coordinates to newer assembly versions, as well as converting coordinates from new versions to older assemblies. Thus, each record may now be associated with multiple genomic coordinates (from multiple assembly versions). For example, since the last version of ORegAnno was published in 2008, the human genome assembly version GRCh38/hg38 was released. All existing ORegAnno human records having genomic coordinates based on assembly versions GRCh36/hg18 or GRCh37/hg19 now have additional updated coordinates using GRCh38/hg38. Similarly, new records that were entered using GRCh38/hg38 coordinates have received additional coordinates based on GRCh37/hg19 and GRCh36/hg18. This allows users to access the genomic coordinates of regulatory regions for the assembly versions that best suit their purposes. Finally, new types of transcriptional regulation have been defined in the current release (Figure 1C and D). These includes microRNA and small non-coding RNA binding sites, as well as operons that function to regulate multiple genes under a single promoter.

### New records

ORegAnno has maintained a focus on incorporating records derived from high quality, manually curated evidence for gene regulation. These typically include experimental evidence demonstrating that binding of a regulatory element to a specific region of DNA or RNA alters corresponding gene expression levels. In total, the current release of ORegAnno contains 2010 unique records covering 112 582 bp derived directly through literature curation, including 661 records that have been added since the previous ORegAnno release.

Highly validated external databases that had been incorporated into earlier ORegAnno releases have been updated. This includes 1874 new records covering an additional 3 591 656 bp derived from VISTA Enhancers (28) (2196 total records covering 3 996 796 total bp), 2934 new records covering an additional 863 201 bp derived from the Yeast Regulatory Map (29) (7320 total records covering 899 449 total bp), as well as 2051 new transcription factor binding site records covering an additional 29 405 bp derived from REDfly (30) (2695 total records covering 913 486 total bp). Previously, ORegAnno had imported records from FlyReg (31), which has since been merged into REDfly.

New records have been created by importing data from external databases that were not found in previous ORegAnno releases. This includes 1 093 443 records covering 11 780 604 bp imported from the JASPAR CORE database (32), which contains a curated, non-redundant set of experimentally obtained transcription factor binding sites in eukaryotes. 783 742 records covering 300 003 052 bp were imported from the PAZAR database (33), which included only records with curated evidence of transcription factor binding and regulatory sequence annotation across various species. 11 451 records covering 4 194 677 bp were derived from RegulonDB (34), a database of transcriptional regulation in *Escherichia coli* K-12, and includes manually curated records that have been complemented with high throughput datasets and comprehensive computational predictions. We combined conserved miRNA target site predictions from miRanda-mirSVR (35) with experimentally-validated miRNA-target interaction data from miRTarBase (36), leading to the addition of 3 072 new ORegAnno records covering 44 353 bp. 131 records covering 1216 bp were derived from NFI-RegulomeDB (37), a database with curated binding sites for the NFI (Nuclear Factor I) family of transcription factors using data from the published literature. Finally, 51 transcription factor binding site records covering 7503 bp were created from the PCNE database of phylogenetically conserved noncoding elements (38).

Because of the open and accessible design of the ORegAnno database and website, ORegAnno has been used for submitting published experimental data. Since the previous ORegAnno release, four datasets derived from high throughput studies have been submitted, and were subsequently curated to ensure that only regulatory regions with a high degree of evidence were retained. These include RELA (p65) ChIP−PET binding sites in human monocytes (39) (489 records covering 52 886 bp), ESR1 binding sites in human MCF-7 breast cancer cells (40) (1234 records covering 165 538 bp), Esr1 binding sites in mouse liver (41) (5568 records covering 2 378 460 bp), and Foxa2 binding sites in mouse liver (7) (11 475 records covering 8 236 933 bp). In all of these cases, DNA sequences were filtered according to signal strength and proximity to signal peak to reduce false positive calls. A summary of the number of records and genomic coverage contributed by each data source is shown in Figure 1E, F and Supplementary Table S1.

### Data access

The ORegAnno database continues to be accessible under an open-source license (GNU Lesser General Public License), in order to encourage development and participation from the community. Monthly ORegAnno database summaries are automatically performed and provide fundamental regulatory information from ORegAnno in a tab-delimited text file that is available for free download, without the need to register with the ORegAnno website (http://www.oreganno.org/).

The ORegAnno website back end code has been updated to improve security and performance, and to accommodate the new data types, dataset sources, and the increased number of records that have been added since the previous release. New search functionality has been added, including the ability to browse records by transcription factor/regulatory element of interest. Source code for the ORegAnno website is available at https://java.net/projects/oreganno/.

The regulatory regions and associated annotation for all supported species have been submitted to the UCSC Genome Browser (14) as updates to existing ORegAnno tracks. This updates existing tracks with a more comprehensive collection of putatively regulatory elements, and additionally provides new tracks on several genome assembly versions.

### Applications

Recently, there has been immense focus on the role of regulatory regions in cancer. In particular, recurrent somatic mutations in the *TERT* promoter have been identified in various cancer types (42–45), and are associated with increased expression of *TERT*. Although the importance of *TERT* up-regulation in cancer has been well-established for nearly two decades (46), it is only in recent years that we've identified the regulatory mechanism driving *TERT* up-regulation in such cases. While additional efforts have identified a small number of other recurrent regulatory mutations in cancer (47–49), this number is far smaller than the recurrent protein-coding mutations that have been identified. This is likely due to several factors, including that most cancer survey projects have focused primarily on coding regions by using exome capture reagents to enrich for these regions, and that the *TERT* promoter region, as with many other genes, has a high GC content making both PCR amplification and sequencing challenging.

**Figure 3.**
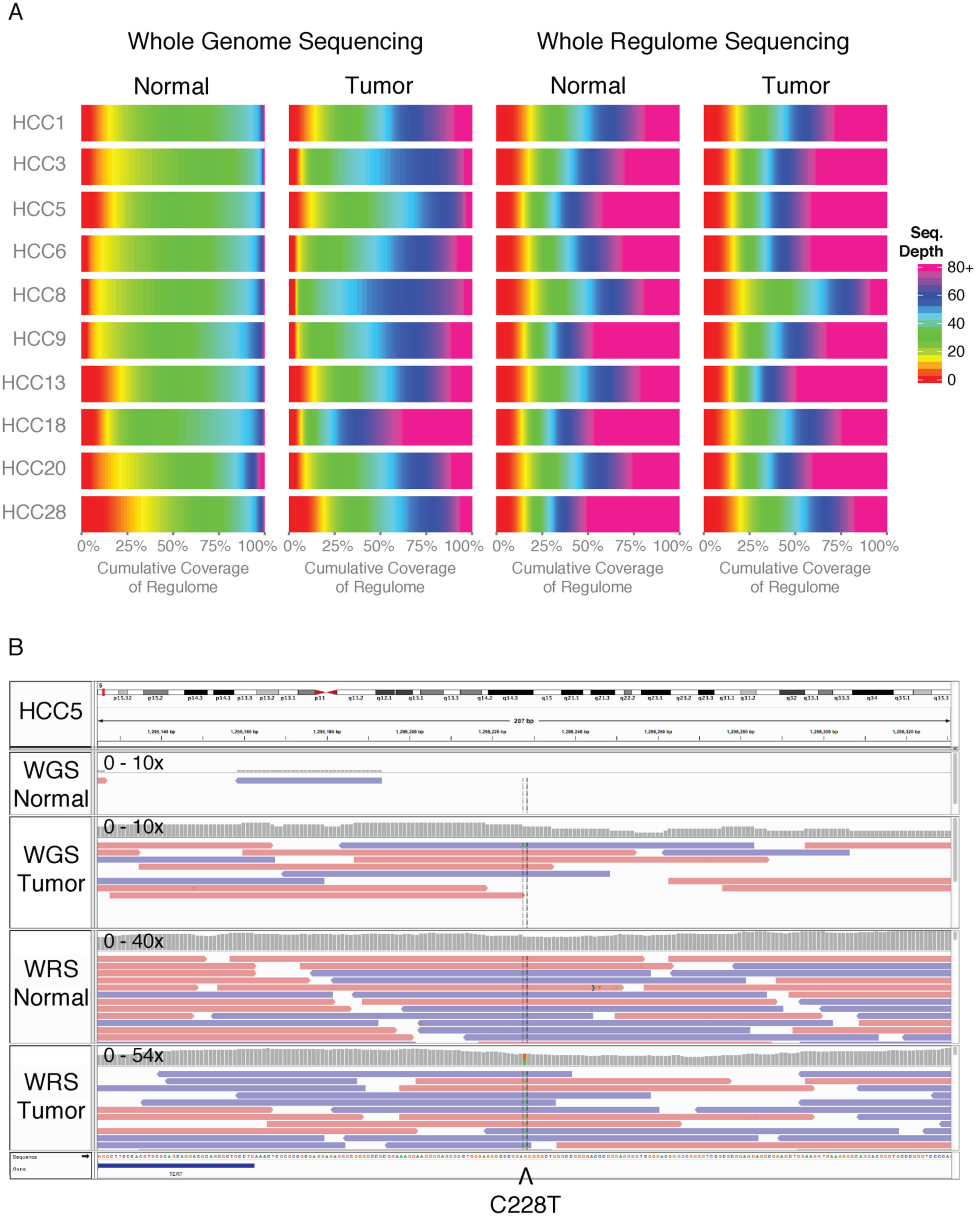
Capture reagent using ORegAnno sites improves coverage of regulatory regions in human hepatocellular carcinoma. (**A**) Coverage across the entire ‘regulome’ is visualized as a heatmap for each of the ten HCC cases. WRS samples have greater sequencing read depth across the targeted region, compared with WGS samples. (**B**) An illustrative IGV (51) screenshot is shown of the *TERT* promoter for one HCC case. A canonical C228T somatic mutation is observed in the WRS data, but cannot be reliably called in the WGS data.

Previous identification of coding regions of the genome made it possible to perform exome targeted sequencing of these regions in a large number of cancer cases at a relatively low cost. Similarly, we've used ORegAnno and other sources to design a ‘regulome’ capture reagent for targeted sequencing. The high quality, relatively small coverage of literature-curated transcription factor binding sites, regulatory polymorphisms, and NFI-RegulomeDB (37) sites identified in ORegAnno, in conjunction with regulatory regions defined by FunSeq (50), and 500 bp regions upstream of each gene transcription start site, were used to define the ‘regulome’ region. As a proof of principle, we then applied ‘regulome’ capture-sequencing to ten normal/tumor pairs of hepatocellular carcinoma (HCC). Overall coverage of the regulatory region defined in the capture reagent was higher in whole regulome sequencing (WRS) samples versus whole genome sequencing (WGS) samples of the same tissues, with median average read depths of 29× in WGS normal, 49× in WGS tumor, 60× in WRS normal and 68× in WRS tumor (Figure 3A, Supplementary Table S2). This improved coverage allowed us to reliably identify the canonical somatic *TERT* promoter mutation C228T in six of the ten cases, an illustrative example of which is shown in Figure 3B.

## References

[1] Montgomery S.B., Griffith O.L., Sleumer M.C., Bergman C.M., Bilenky M., Pleasance E.D., Prychyna Y., Zhang X., Jones S.J. (2006). ORegAnno: an open access database and curation system for literature-derived promoters, transcription factor binding sites and regulatory variation. Bioinformatics.

[2] Griffith O.L., Montgomery S.B., Bernier B., Chu B., Kasaian K., Aerts S., Mahony S., Sleumer M.C., Bilenky M., Haeussler M. (2008). ORegAnno: an open-access community-driven resource for regulatory annotation. Nucleic Acids Res..

[3] The Encode Project Consortium (2012). An integrated encyclopedia of DNA elements in the human genome. Nature.

[4] Doolittle W.F. (2013). Is junk DNA bunk? A critique of ENCODE. Proc. Natl. Acad. Sci. U.S.A..

[5] Eddy S.R. (2013). The ENCODE project: missteps overshadowing a success. Curr. Biol..

[6] Graur D., Zheng Y., Price N., Azevedo R.B., Zufall R.A., Elhaik E. (2013). On the immortality of television sets: ‘function’ in the human genome according to the evolution-free gospel of ENCODE. Genome Biol. Evol..

[7] Wederell E.D., Bilenky M., Cullum R., Thiessen N., Dagpinar M., Delaney A., Varhol R., Zhao Y., Zeng T., Bernier B. (2008). Global analysis of in vivo Foxa2-binding sites in mouse adult liver using massively parallel sequencing. Nucleic Acids Res..

[8] Medina I., Carbonell J., Pulido L., Madeira S.C., Goetz S., Conesa A., Tarraga J., Pascual-Montano A., Nogales-Cadenas R., Santoyo J. (2010). Babelomics: an integrative platform for the analysis of transcriptomics, proteomics and genomic data with advanced functional profiling. Nucleic Acids Res..

[9] Sleumer M.C., Bilenky M., He A., Robertson G., Thiessen N., Jones S.J. (2009). Caenorhabditis elegans cisRED: a catalogue of conserved genomic elements. Nucleic Acids Res..

[10] Hooghe B., Hulpiau P., van Roy F., De Bleser P. (2008). ConTra: a promoter alignment analysis tool for identification of transcription factor binding sites across species. Nucleic Acids Res..

[11] Eicher J.D., Landowski C., Stackhouse B., Sloan A., Chen W., Jensen N., Lien J.P., Leslie R., Johnson A.D. (2015). GRASP v2.0: an update on the Genome-Wide Repository of Associations between SNPs and phenotypes. Nucleic Acids Res..

[12] Imrichova H., Hulselmans G., Atak Z.K., Potier D., Aerts S. (2015). i-cisTarget 2015 update: generalized cis-regulatory enrichment analysis in human, mouse and fly. Nucleic Acids Res..

[13] Lee C., Huang C.H. (2014). LASAGNA-Search 2.0: integrated transcription factor binding site search and visualization in a browser. Bioinformatics.

[14] Rosenbloom K.R., Armstrong J., Barber G.P., Casper J., Clawson H., Diekhans M., Dreszer T.R., Fujita P.A., Guruvadoo L., Haeussler M. (2015). The UCSC Genome Browser database: 2015 update. Nucleic Acids Res..

[15] Baitaluk M., Kozhenkov S., Ponomarenko J. (2012). An integrative approach to inferring gene regulatory module networks. PLoS One.

[16] Chu G.C., Zhau H.E., Wang R., Rogatko A., Feng X., Zayzafoon M., Liu Y., Farach-Carson M.C., You S., Kim J. (2014). RANK- and c-Met-mediated signal network promotes prostate cancer metastatic colonization. Endocr. Relat. Cancer.

[17] Fazekas D., Koltai M., Turei D., Modos D., Palfy M., Dul Z., Zsakai L., Szalay-Beko M., Lenti K., Farkas I.J. (2013). SignaLink 2 - a signaling pathway resource with multi-layered regulatory networks. BMC Syst. Biol..

[18] Komurov K., White M.A., Ram P.T. (2010). Use of data-biased random walks on graphs for the retrieval of context-specific networks from genomic data. PLoS Comput. Biol..

[19] Turei D., Foldvari-Nagy L., Fazekas D., Modos D., Kubisch J., Kadlecsik T., Demeter A., Lenti K., Csermely P., Vellai T. (2015). Autophagy Regulatory Network - a systems-level bioinformatics resource for studying the mechanism and regulation of autophagy. Autophagy.

[20] Bainbridge M.N., Wang M., Wu Y., Newsham I., Muzny D.M., Jefferies J.L., Albert T.J., Burgess D.L., Gibbs R.A. (2011). Targeted enrichment beyond the consensus coding DNA sequence exome reveals exons with higher variant densities. Genome Biol..

[21] Griffith M., Griffith O.L., Smith S.M., Ramu A., Callaway M.B., Brummett A.M., Kiwala M.J., Coffman A.C., Regier A.A., Oberkfell B.J. (2015). Genome Modeling System: A Knowledge Management Platform for Genomics. PLoS Comput. Biol..

[22] Baca S.C., Prandi D., Lawrence M.S., Mosquera J.M., Romanel A., Drier Y., Park K., Kitabayashi N., MacDonald T.Y., Ghandi M. (2013). Punctuated evolution of prostate cancer genomes. Cell.

[23] Li M.J., Wang L.Y., Xia Z., Sham P.C., Wang J. (2013). GWAS3D: Detecting human regulatory variants by integrative analysis of genome-wide associations, chromosome interactions and histone modifications. Nucleic Acids Res..

[24] Stransky N., Egloff A.M., Tward A.D., Kostic A.D., Cibulskis K., Sivachenko A., Kryukov G.V., Lawrence M.S., Sougnez C., McKenna A. (2011). The mutational landscape of head and neck squamous cell carcinoma. Science.

[25] Turnbull C., Perdeaux E.R., Pernet D., Naranjo A., Renwick A., Seal S., Munoz-Xicola R.M., Hanks S., Slade I., Zachariou A. (2012). A genome-wide association study identifies susceptibility loci for Wilms tumor. Nat. Genet..

[26] Zerbino D.R., Wilder S.P., Johnson N., Juettemann T., Flicek P.R. (2015). The ensembl regulatory build. Genome Biol..

[27] Kent W.J., Baertsch R., Hinrichs A., Miller W., Haussler D. (2003). Evolution's cauldron: duplication, deletion, and rearrangement in the mouse and human genomes. Proc. Natl. Acad. Sci. U.S.A..

[28] Visel A., Minovitsky S., Dubchak I., Pennacchio L.A. (2007). VISTA Enhancer Browser–a database of tissue-specific human enhancers. Nucleic Acids Res..

[29] MacIsaac K.D., Wang T., Gordon D.B., Gifford D.K., Stormo G.D., Fraenkel E. (2006). An improved map of conserved regulatory sites for Saccharomyces cerevisiae. BMC Bioinformatics.

[30] Gallo S.M., Gerrard D.T., Miner D., Simich M., Soye B., Bergman C.M., Halfon M.S. (2011). REDfly v3.0: toward a comprehensive database of transcriptional regulatory elements in Drosophila. Nucleic Acids Res..

[31] Bergman C.M., Carlson J.W., Celniker S.E. (2005). Drosophila DNase I footprint database: a systematic genome annotation of transcription factor binding sites in the fruitfly, Drosophila melanogaster. Bioinformatics.

[32] Mathelier A., Zhao X., Zhang A.W., Parcy F., Worsley-Hunt R., Arenillas D.J., Buchman S., Chen C.Y., Chou A., Ienasescu H. (2014). JASPAR 2014: an extensively expanded and updated open-access database of transcription factor binding profiles. Nucleic Acids Res..

[33] Portales-Casamar E., Arenillas D., Lim J., Swanson M.I., Jiang S., McCallum A., Kirov S., Wasserman W.W. (2009). The PAZAR database of gene regulatory information coupled to the ORCA toolkit for the study of regulatory sequences. Nucleic Acids Res..

[34] Salgado H., Peralta-Gil M., Gama-Castro S., Santos-Zavaleta A., Muniz-Rascado L., Garcia-Sotelo J.S., Weiss V., Solano-Lira H., Martinez-Flores I., Medina-Rivera A. (2013). RegulonDB v8.0: omics data sets, evolutionary conservation, regulatory phrases, cross-validated gold standards and more. Nucleic Acids Res..

[35] Betel D., Koppal A., Agius P., Sander C., Leslie C. (2010). Comprehensive modeling of microRNA targets predicts functional non-conserved and non-canonical sites. Genome Biol..

[36] Hsu S.D., Tseng Y.T., Shrestha S., Lin Y.L., Khaleel A., Chou C.H., Chu C.F., Huang H.Y., Lin C.M., Ho S.Y. (2014). miRTarBase update 2014: an information resource for experimentally validated miRNA-target interactions. Nucleic Acids Res..

[37] Gronostajski R.M., Guaneri J., Lee D.H., Gallo S.M. (2011). The NFI-Regulome Database: A tool for annotation and analysis of control regions of genes regulated by Nuclear Factor I transcription factors. J. Clin. Bioinforma.

[38] Hufton A.L., Mathia S., Braun H., Georgi U., Lehrach H., Vingron M., Poustka A.J., Panopoulou G. (2009). Deeply conserved chordate noncoding sequences preserve genome synteny but do not drive gene duplicate retention. Genome Res..

[39] Lim C.A., Yao F., Wong J.J., George J., Xu H., Chiu K.P., Sung W.K., Lipovich L., Vega V.B., Chen J. (2007). Genome-wide mapping of RELA(p65) binding identifies E2F1 as a transcriptional activator recruited by NF-kappaB upon TLR4 activation. Mol. Cell.

[40] Lin C.Y., Vega V.B., Thomsen J.S., Zhang T., Kong S.L., Xie M., Chiu K.P., Lipovich L., Barnett D.H., Stossi F. (2007). Whole-genome cartography of estrogen receptor alpha binding sites. PLoS Genet..

[41] Gao H., Falt S., Sandelin A., Gustafsson J.A., Dahlman-Wright K. (2008). Genome-wide identification of estrogen receptor alpha-binding sites in mouse liver. Mol. Endocrinol..

[42] Horn S., Figl A., Rachakonda P.S., Fischer C., Sucker A., Gast A., Kadel S., Moll I., Nagore E., Hemminki K. (2013). TERT promoter mutations in familial and sporadic melanoma. Science.

[43] Huang F.W., Hodis E., Xu M.J., Kryukov G.V., Chin L., Garraway L.A. (2013). Highly recurrent TERT promoter mutations in human melanoma. Science.

[44] Killela P.J., Reitman Z.J., Jiao Y., Bettegowda C., Agrawal N., Diaz L.A. Jr, Friedman A.H., Friedman H., Gallia G.L., Giovanella B.C. (2013). TERT promoter mutations occur frequently in gliomas and a subset of tumors derived from cells with low rates of self-renewal. Proc. Natl. Acad. Sci. U.S.A..

[45] Vinagre J., Almeida A., Populo H., Batista R., Lyra J., Pinto V., Coelho R., Celestino R., Prazeres H., Lima L. (2013). Frequency of TERT promoter mutations in human cancers. Nat. Commun..

[46] Zhang X., Mar V., Zhou W., Harrington L., Robinson M.O. (1999). Telomere shortening and apoptosis in telomerase-inhibited human tumor cells. Genes Dev..

[47] Fredriksson N.J., Ny L., Nilsson J.A., Larsson E. (2014). Systematic analysis of noncoding somatic mutations and gene expression alterations across 14 tumor types. Nat. Genet..

[48] Melton C., Reuter J.A., Spacek D.V., Snyder M. (2015). Recurrent somatic mutations in regulatory regions of human cancer genomes. Nat. Genet..

[49] Weinhold N., Jacobsen A., Schultz N., Sander C., Lee W. (2014). Genome-wide analysis of noncoding regulatory mutations in cancer. Nat. Genet..

[50] Khurana E., Fu Y., Colonna V., Mu X.J., Kang H.M., Lappalainen T., Sboner A., Lochovsky L., Chen J., Harmanci A. (2013). Integrative annotation of variants from 1092 humans: application to cancer genomics. Science.

[51] Thorvaldsdottir H., Robinson J.T., Mesirov J.P. (2013). Integrative Genomics Viewer (IGV): high-performance genomics data visualization and exploration. Brief Bioinform..

